# A male with primary accessory breast carcinoma in an axilla is strongly suspected of having hereditary breast cancer

**DOI:** 10.1007/s13691-020-00466-8

**Published:** 2021-01-10

**Authors:** Eriko Takahashi, Kaori Terata, Hiroshi Nanjo, Koichi Ishiyama, Yuko Hiroshima, Ayuko Yamaguchi, Misako Yatsuyanagi, Chiaki Kudo, Akiyuki Wakita, Shinogu Takashima, Yusuke Sato, Kazuhiro Imai, Satoru Motoyama, Yoshihiro Minamiya

**Affiliations:** 1 Department of Thoracic Surgery, Akita University Graduate School of Medicine, 1-1-1 Hondo, Akita, 010-8543 Japan; 2 Department of Pathology, Akita University Hospital, Akita, Japan; 3 Department of Diagnostic Radiology, Akita University Hospital, Akita, Japan

**Keywords:** Accessory breast cancer, Male breast cancer, Hereditary breast and ovarian cancer syndrome, BRCA genetic testing, Hereditary breast cancer genes

## Abstract

We herein report on a male with primary accessory breast cancer in an axilla. A 75-year-old man first noticed a subcutaneous nodule about 2 cm in diameter in the area of his right axilla. The patient underwent extirpation of the mass in a public hospital. Histological examination revealed invasive breast carcinoma of no special type associated with mucinous carcinoma, invasive micropapillary carcinoma and intraductal components. Immunohistochemical analysis showed that the tumor cells were positive for Gross cystic disease fluid protein (GCDFP)-15, mammaglobin and GATA3. Staining for estrogen receptor (ER) and progesterone receptor (PR) was positive, and human epidermal growth factor receptor 2 (HER2) was negative. The Ki67 labeling index (LI) was 33.6%. Imaging revealed no evidence of a primary tumor in any other organ or in the bilateral mammary gland. We performed radical resection of the right axilla, including the scar, and axillary lymph node dissection. The final pathological examination of the surgical specimen showed normal mammary gland tissue that was not connected to the proper mammary gland, and no residual cancer or metastatic lymph nodes. Based on our clinical and pathological findings, this tumor was diagnosed as breast cancer originating from the accessory mammary gland in the right axilla. After surgery, tamoxifen was administered as adjuvant therapy. Since the surgery, 2 years ago, there has been no evidence of recurrence. Hereditary Breast and Ovarian Cancer syndrome was suspected in this case because the patient was a male with breast cancer, and he had two first-degree relatives with breast cancer. This patient had no BRCA mutations on genetic testing. Nonetheless, in cases of male breast cancer, it is necessary to obtain genetic information due to the possibility of hereditary breast cancer, including cancers associated with BRCA gene mutation.

## Introduction

Breast carcinoma in males accounts for just 0.6% of all malignant breast neoplasm cases in Japan [1]. The total incidence rate of accessory breast carcinoma is 0.2–0.6% [2], and males account for only 2.4–5.3% of accessory breast carcinoma cases, making it rare [3]. In cases of male breast cancer, it is important to consider the potential for hereditary breast cancer, including cancers associated with BRCA gene mutation. Here, we report the case of a male with primary accessory breast carcinoma in an axilla and consider the possibility of hereditary breast cancer.

## Case report

A 75-year-old man first noticed a subcutaneous nodule about 2 cm in diameter in the area of the right axilla and consulted a public hospital. He was palpated and given a probable diagnosis of a benign tumor. The patient then underwent extirpation of the mass under local anesthesia. The pathological diagnosis of the resected sample was primary breast cancer of the axillary accessory mammary gland. The patient was then referred to our hospital.

**Fig. 1:**
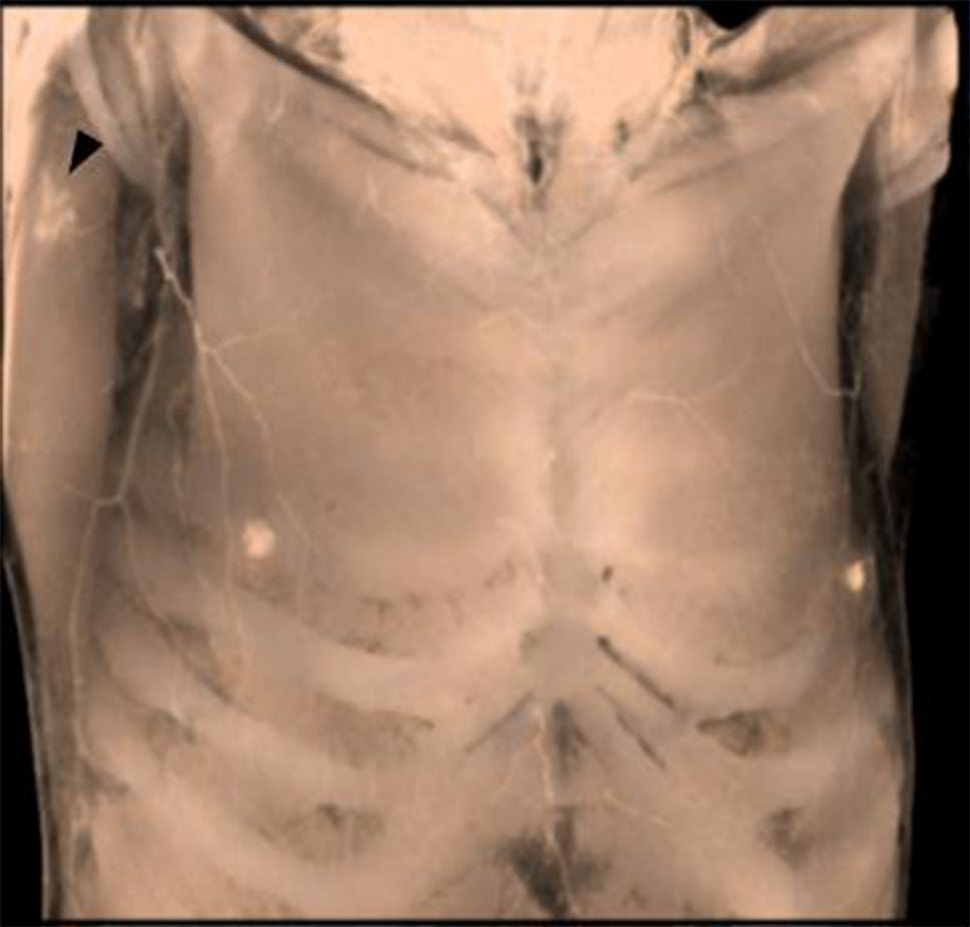
A 3D image of the CT after excisional biopsy. A scar was present within the axilla but outside the normal milk line (arrowhead)

**Fig. 2:**
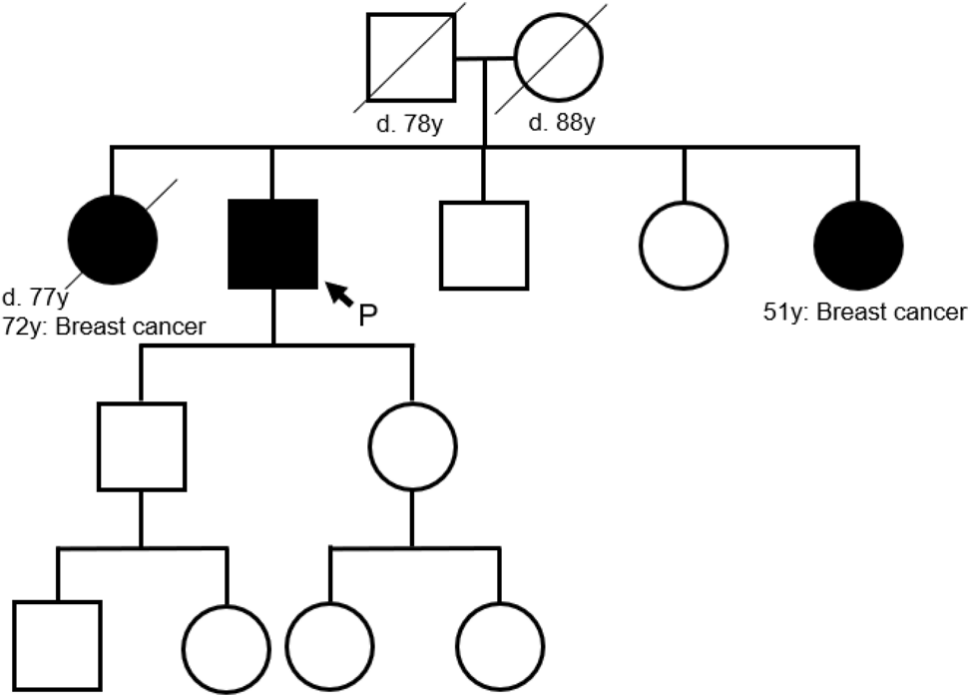
Family tree. The patient’s older and younger sisters developed breast cancer at age 72 and 51 years, respectively

At the initial visit to our hospital, physical examination showed only a surgical scar with no mass near the right axillary area. A scar was present within the axilla but outside the milk line (Fig. 1). The bilateral breasts had no palpable mass, and there were also no significant palpable lymph nodes in the bilateral axillae or upper and lower clavicles. The family’s history showed that the patient’s older and younger sisters developed breast cancer at the ages of 72 and 51, respectively (Fig. 2).

**Fig. 3:**
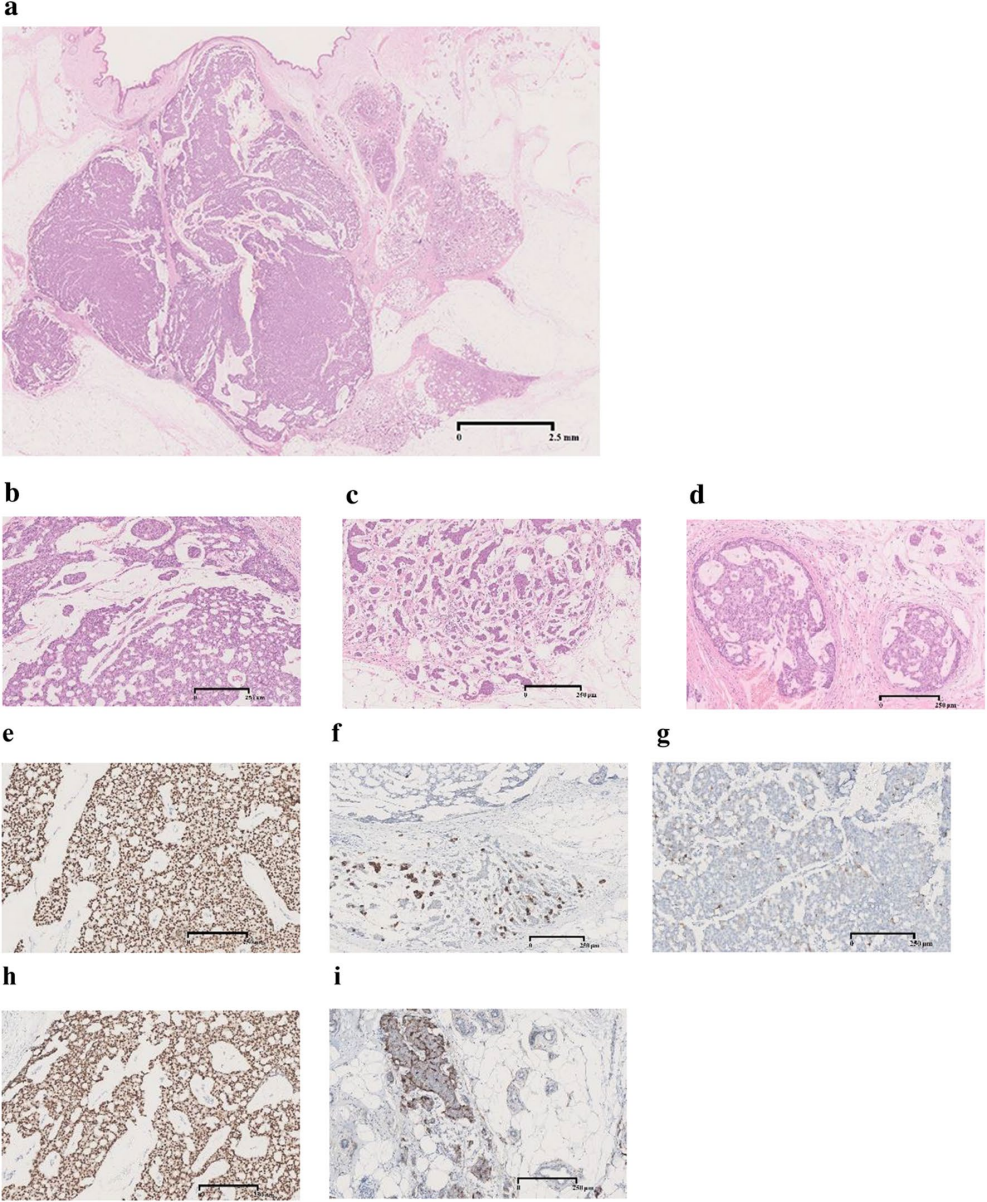
Pathological examination of excised tumor.** a** The tumor was 13 × 11 mm in diameter. There was no involvement of skin or skeletal muscle. Histologic features were proliferation of atypical ductal cells. The cells had enlarged and irregular nuclei, infiltrated fat tissue and formed tubular structures, mostly indicating invasive breast carcinoma of no special type. **b** The tumor contained mucinous carcinoma components occupying 20% of its area. **c** The tumor contained invasive micropapillary components occupying 10% of its area. **d** The majority of the carcinoma area was invasive breast carcinoma, though a few intraductal components were also present. **e** GATA3 was diffusely positive. **f** Mammaglobin was positive in a portion of cells. **g** GCDFP-15 was positive in a portion of cells. **h** ER was diffusely positive. **i** CD10 was positive in intraductal components

The excised tumor was 13 × 11 mm in diameter. There was no involvement of skin or skeletal muscle. Histological assessment revealed invasive breast carcinoma of no special type associated with mucinous and invasive micropapillary carcinoma. The mucinous and invasive micropapillary carcinoma components occupied 20% and 10% of the tumor area, respectively. The majority of the carcinoma area was invasive breast carcinoma, though a few intraductal components were also present, as confirmed by immunostaining for p65, CD10 and αSMA. Immunostaining for D2–40 and CD31 and elastica-Masson staining revealed infiltration of fat tissue, without vascular or lymphatic invasion. Immunohistochemical analysis showed that the tumor cells were positive for E-cadherin, Gross cystic disease fluid protein (GCDFP)-15, mammaglobin and GATA3, and were negative for synaptophysin. Staining for estrogen receptor (ER) and progesterone receptor (PR) was positive in 98% of the tumor cell nuclei, and human epidermal growth factor receptor 2 (HER2) was negative in the tumor cells. Ki-67 labeling index (LI) was 33.6% (Fig. 3).

Tumor markers were within the normal range (CEA 2.5 ng/ml, CA15-3 10.7 U/ml, NCC-ST-439 < 1.0 U/ml).

**Fig. 4:**
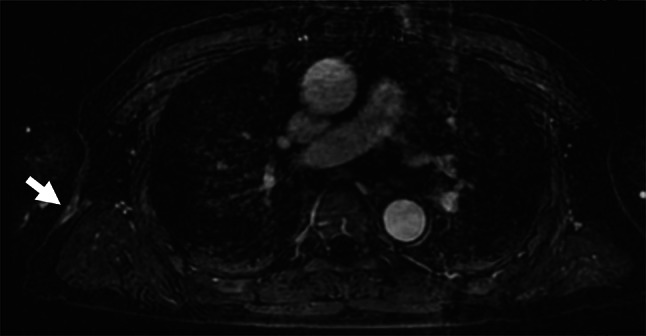
After extirpation of the mass, MRI showed only slight enhancement at the scar site, with no significant local residual tumor (arrow)

Computed tomography, magnetic resonance imaging and ultrasonography showed the following: (1) slight enhancement that appeared to be changed after the tumor resection, with no significant local residual tumor (Fig. 4); (2) no primary lesion in the bilateral mammary glands; (3) no swelling of bilateral axillary lymph nodes; (4) no obvious signs of a malignant primary tumor in another part of the body.

Based on the clinical and pathological findings, this tumor was diagnosed as breast cancer originating from an accessory mammary gland in the right axilla, rather than a cutaneous adnexal carcinoma.

We performed radical resection of the right axilla, including the scar from the excisional biopsy, and axillary lymph node dissection under general anesthesia.

**Fig. 5:**
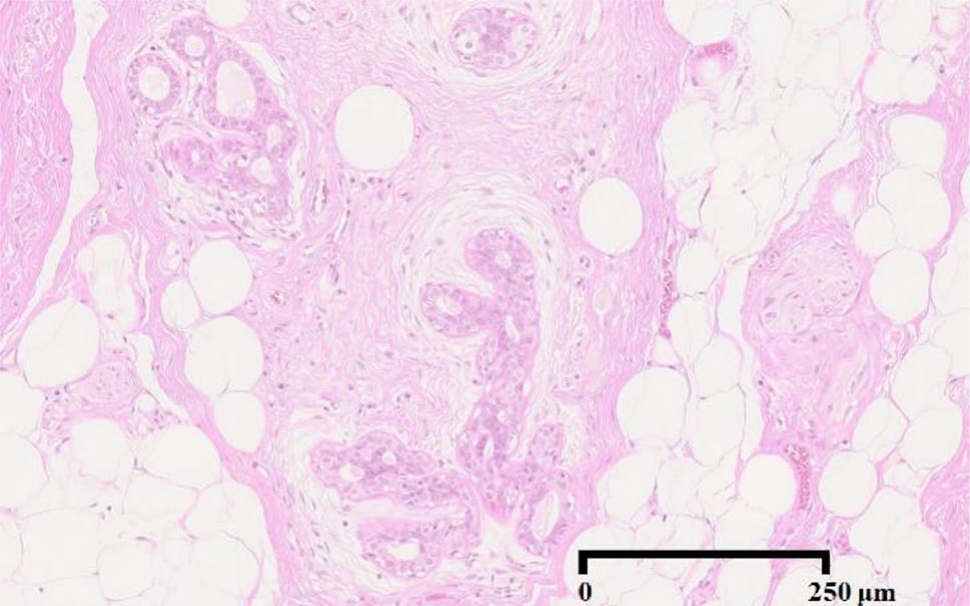
The final pathological examination of the surgical specimen showed normal mammary gland tissue that was not connected to the proper mammary gland. There was no residual cancer

The final pathological examination of a surgical specimen showed normal mammary gland tissue that was not connected to the proper mammary gland and postoperative fibrotic scarring. No residual cancer or metastatic lymph nodes were found (Fig. 5).

Postoperative clinical course: the patient recovered uneventfully and was discharged 10 days postoperatively. Tamoxifen at 20 mg/day was administered as adjuvant therapy. The patient has presented no metastatic lesions in the 2 years since the operation and has been receiving hormone therapy. Because there was the possibility that Hereditary Breast and Ovarian Cancer syndrome (HBOC) was the cause of this case of male breast cancer, given that the patient has two first-degree relatives with breast cancer, the patient received genetic counseling after his surgery. Initially, the patient declined genetic testing, but 2 years after his operation, he requested it. However, no clinically significant BRCA1/2 gene mutation was identified.

## Discussion

**Fig. 6:**
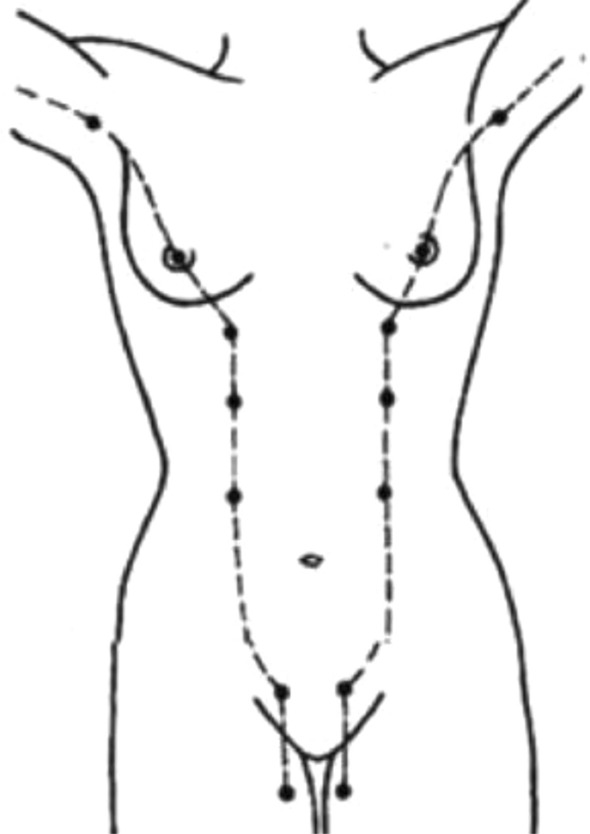
Milk line

Accessory breast carcinoma in males is rare [1–3]. In addition to Maki et al.’s compilation of Japanese reports from 1996 to 2013 [4], we performed a journal search using “Men/accessory Breast Cancer” as keywords and found that from January 2014 to June 2020, only 26 cases of accessory breast cancer in males were reported in Japan. Extra or accessory breasts usually occur on the milk line (Fig. 6) [5]. In this case, the tumor was present within the axilla but outside the milk line, so preoperative diagnosis of accessory breast cancer was very difficult.

It is often difficult to distinguish between breast cancer and cancer derived from sweat glands or sebaceous glands, and the presence of carcinoma in situ or ER/PR positivity is used for differentiation [6]. Additionally, accessory breast cancer must be pathologically demonstrated to be located adjacent to normal breast ducts or lobules that are not connected to the proper mammary gland, and it is also necessary to exclude the possibility of a metastatic lesion from another primary cancer [7]. In our case, we made a diagnosis of accessory breast carcinoma of the axilla because there was no evidence of other organ cancer and mammary gland tissue discontinuous with the normal breast was present. The majority of the tumor was invasive carcinoma, though a few intraductal components were also present. The tumor cells of the invasive carcinoma were positive for GCDFP-15, mammaglobin, GATA3, ER and PR.

According to data compiled by Maki et al., local radical resection and axillary lymph node dissection is the most common surgical approach, accounting for 64.0% of surgical treatments [4]. In our case, no lesions were found in the bilateral breasts, and we performed radical resection of the right axilla, including the biopsy scar, and axillary lymph node dissection. Postoperative pathology showed no axillary lymph node metastasis, indicating it would have been possible to avoid dissection. Whether or not sentinel lymph node biopsy is an appropriate approach in cases of accessory breast cancer is an issue to be addressed in the future. In our case, sentinel lymph node biopsy was considered to be contraindicated because lymph flow may have been inhibited after the excisional biopsy.

In male breast cancer, adjuvant chemotherapy is recommended adheres to guidelines used for women and Tamoxifen is recommended for adjuvant endocrine therapy [8]. In our case, considering low risk of recurrence, tamoxifen was administered as adjuvant therapy.

Guidelines such as the “NCCN Guideline Genetic/Familial High-Risk Assessment: Breast and Ovarian 2019 ver3 [9].” and the “Guidebook for Diagnosis and Treatment of Hereditary Breast and Ovarian Cancer Syndrome 2017[10]” point out the importance of always considering the potential for HBOC in cases of male breast cancer. Since April 2020, BRCA genetic testing has been covered by insurance, which reaffirms the importance of HBOC. Additionally, coverage by insurance is expected to increase the proportion of patients undergoing BRCA genetic testing in the future. It is expected that this will enable clarification of the BRCA gene mutation rate in male breast cancer. HBOC was suspected in this case because the patient was a male with breast cancer, and he had two first-degree relatives with breast cancer. The patient initially declined a genetic test, but two years later, after treatment was complete and the BRCA genetic test was covered by insurance in Japan, he requested it. The patient now has two children and four grandchildren, and he was motivated by the potential genetic impact on the next generation. Our patient had no BRCA mutations but given his family history, we cannot rule out the possibility of hereditary breast cancer genes other than BRCA. It also cannot be concluded that his relatives, who have not been tested, do not carry hereditary breast cancer genes, including BRCA. Genetic testing of this patient’s relatives who developed breast cancer may be important for the blood relatives around them.

In addition to BRCA 1/2, associations between breast cancer risk and protein-truncating variants of PALB2, CHEK2, ATM, TP53, PTEN, NF1, Double carrier, CDH1 and NBN have been established [11]. Therefore, multigene panel testing to identify gene mutations other than BRCA may be useful. However, few of the mutations identified so far have been shown to correlate with actual risk. Moreover, mutations in variants of uncertain significance have also been identified with high probability. Therefore, consideration of these is currently not recommended [10].

Physicians and patients lack awareness of male accessory breast cancer because it is rare. Tumors that develop on and around the milk line should always be carefully examined, given the potential for accessory breast cancer. Additionally, in cases of male breast cancer, it is necessary to obtain genetic information due to the possibility of hereditary breast cancer, including cancers associated with BRCA gene mutation.
